# Structural and functional benchmarking of monolayer- and bioreactor-generated hiPSC-derived cardiomyocytes

**DOI:** 10.1063/5.0335477

**Published:** 2026-07-09

**Authors:** Yongjun Jang, Kevin Shani, Anna Clouvel-Gervaiseau, Maksymilian Prondzynski, Simone Nuebling, Christopher J. Shin, Yichong Wang, Giulio Ciucci, Michio Kawai, Yoonseo Lee, Griffin Radtke, Durgesh Bonde, William T. Pu, Kevin Kit Parker

**Affiliations:** 1 Disease Biophysics Group, John A. Paulson School of Engineering and Applied Sciences, Harvard University, Boston, Massachusetts 02134, USA; 2 Department of Cardiology, Boston Children's Hospital, Boston, Massachusetts 02115, USA; 3 Harvard Medical School, Boston, Massachusetts 02115, USA; 4 Harvard Stem Cell Institute, Cambridge, Massachusetts 02138, USA

## Abstract

Transitioning from animal to human cell sources represents a critical milestone in cardiac tissue engineering and biomedical research. Neonatal rat ventricular myocytes (NRVMs) have long served as the functional benchmark for engineered cardiac tissues; however, their rodent origin limits clinical relevance. Human-induced pluripotent stem cell-derived cardiomyocytes (hiPSC-CMs) offer a renewable, species-specific alternative but remain restricted by immature structure and function, small-scale yield, and high batch variability by conventional two-dimensional monolayer (2D-Mono) differentiation. Here, we systematically evaluated hiPSC-CMs generated by 2D-Mono and three-dimensional embryoid-body (3D-EB) differentiation using identical 15-day Wnt-modulated protocols without additional maturation steps. The 3D-EB method yielded 181 × 10^6^ cells per 100 ml, approximately 2.7-fold higher than the 2D-Mono, while maintaining >80% cTnT^+^ purity and reduced batch variability. Structural analyses revealed improved sarcomeric organization in 3D-EB tissues compared with 2D-Mono, although both remained less organized than NRVMs, while other morphological parameters were comparable between groups. Functionally, 3D-EB tissues exhibited faster calcium conduction (33.7 cm/s), indicating enhanced electrical coupling relative to 2D-Mono. Although contractile performance remained similar between differentiation formats and below NRVM levels, 3D-EB tissues exhibited consistent structural and functional improvement in calcium wave velocity and contractility over time. These results show that, even without external maturation cues, 3D-EB differentiation yields reproducible, scalable, and human-relevant cardiomyocytes.

## INTRODUCTION

Cardiovascular disease (CVD) remains the leading cause of death worldwide and places an increasingly heavy burden on healthcare systems.^1^ This growing burden highlights the need for human-relevant heart models that can enhance understanding of disease mechanisms while accelerating drug discovery and translational research. For decades, neonatal rat ventricular myocytes (NRVMs) have remained a widely used cell source for *in vitro* cardiac tissue engineering owing to their experimental robustness.^2,3^ Their relevance has been reinforced by well-characterized rat cardiovascular disease models, such as the spontaneously hypertensive rat, which provide a framework for interpreting *in vitro* findings within the context of maladaptive cardiac remodeling *in vivo*.^4,5^ Nevertheless, fundamental interspecies differences limit their translational applicability.^6^ For example, human ventricular myocytes beat at 60–80 bpm with long, plateaued action potentials, whereas rat myocytes beat at 250–450 bpm with short, rapidly repolarizing action potentials (APs) driven by different potassium currents.^7,8^ Rats also exhibit faster calcium cycling and distinct ion-channel and G-protein-coupled receptor (GPCR) expression profiles that alter electrophysiological responses and drug sensitivity, limiting the ability of NRVMs to accurately model human cardiac behavior.^9–11^ In contrast, human-induced pluripotent stem cell-derived cardiomyocytes (hiPSC-CMs) represent a human-based platform for studying cardiac physiology and disease.^12,13^ While they hold promise for generating patient-specific models, the relationship between genetic variants and functional phenotypes remains poorly understood.^14^

Recently, the development of hiPSC-CMs has promised a reproducible and physiologically relevant cell source for studying human cardiac biology. These cells not only offer the potential to replace animal-derived cardiomyocytes but also provide new opportunities for investigating genetic and acquired heart diseases, personalized drug screening, and regenerative medicine.^15^ In parallel, differentiation protocols based on sequential Wnt pathway activation and inhibition in two-dimensional (2D) culture have been refined, enabling the production of high-purity cardiomyocytes.^16,17^ Despite these advances, 2D-derived hiPSC-CMs remain limited by their small-scale yield and labor-intensive handling, restricting their application in large-scale or multi-platform studies.^18^ To overcome these challenges, three-dimensional (3D) suspension differentiation methods have been developed to support high-density culture and efficient large-scale cardiomyocyte generation.^19,20^ These systems have achieved substantial progress in scalable human cardiac cell production, yielding more than 10^6^ cells per milliliter with over 90% cTnT^+^ purity.^18–20^ In addition, hiPSC-CMs generated in suspension bioreactor systems have been characterized at the molecular and metabolic levels, demonstrating predominantly ventricular identity, enhanced mitochondrial function, and reduced batch-to-batch variability compared to 2D-derived hiPSC-CMs.^18^ Nevertheless, hiPSC-CMs have not yet been systematically benchmarked against NRVMs, a widely used reference standard, in heart-on-chip platforms. Therefore, elucidating and validating the structural and functional characteristics of hiPSC-CMs compared to NRVMs is a critical step toward building multiscale human-based cardiac tissue models that better recapitulate the structure–function relationships of the native human myocardium.

Here, we present a systematic comparison of conventional 2D monolayer (2D-Mono) culture and 3D embryoid-body (3D-EB) suspension culture hiPSC-CMs, differentiated using an identical Wnt-modulated protocol in both formats, standardizing conditions that were previously format-dependent, with NRVMs as a reference standard.^18^ Each cell source was evaluated under identical conditions to compare its structural and functional characteristics. Myofibrillar organization was quantified using orientation order parameter (OOP) analysis, calcium wave velocity (Ca^2+^V) was measured using geometrically insulated (G-node) chips, and contractile stress was assessed by muscular thin films (MTFs). For systematic benchmarking, hiPSC-CMs were assessed at defined culture times (7 and 14 days), while NRVMs served as the established gold-standard reference at day 7. This study provides quantitative reference data and an evaluation framework that enables the transition from traditional NRVM-dependent systems to hiPSC-CM-based engineered cardiac tissues for human-relevant translational research.

## RESULTS AND DISCUSSION

### Establishing consistent and scalable hiPSC-CM production

Three cardiomyocyte populations were examined in this study: NRVMs and hiPSC-derived cardiomyocytes generated via 2D-Mono or 3D-EB differentiation. NRVMs served as the primary cardiomyocyte benchmark across all structural and functional assays and were isolated using a standard protocol previously established in our laboratory.^21,22^

**FIG. 1.**
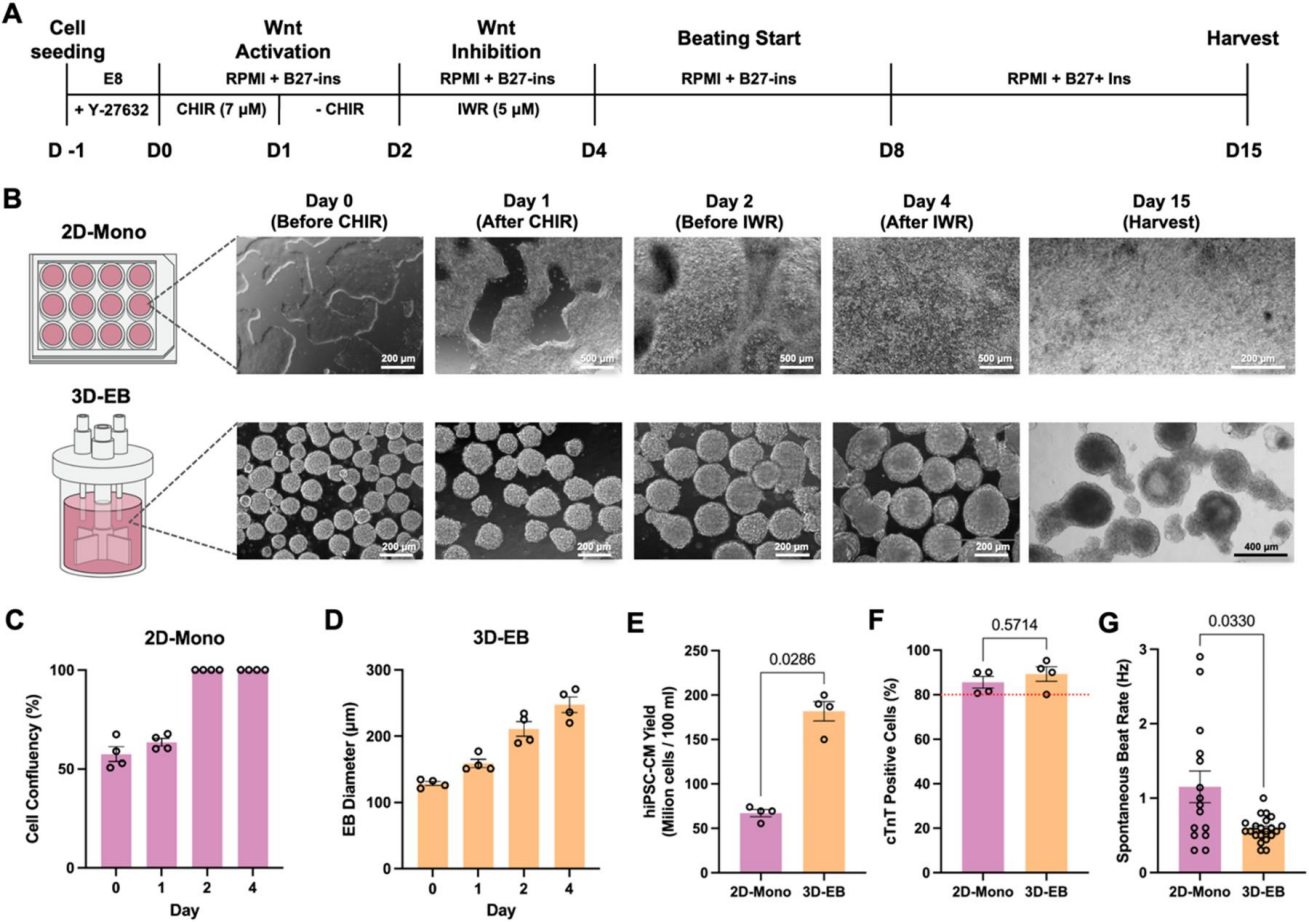
Comparison of 2D-Mono and 3D-EB hiPSC-CM differentiation and quality control. (a) Schematic representation of the Wnt-modulated differentiation timeline used to generate cardiomyocytes from hiPSCs. (b) Representative bright-field images showing the progression of hiPSC-CM differentiation in 2D-Mono and 3D-EB formats at indicated time points. (c) and (d) Quantification of 2D confluency and 3D EB diameter at the onset of differentiation. (e) Flow-cytometric quantification of cTnT^+^ cardiomyocytes. (f) Comparison of total cell yield per differentiation cycle normalized to 100 ml of culture medium. (g) Fraction of spontaneously beating cultures across all batches for 2D and 3D differentiation. Each dot in (c)–(f) represents an independent batch (four batches per group), and each dot in (g) represents an individual well (2D; *n* = 15 from four batches) or EB (3D; *n* = 21 from four batches). Values are presented as mean ± SEM. Statistical significance was evaluated using the non-parametric Mann–Whitney test; *P* values are indicated above each comparison.

To enable a standardized comparison between 2D-Mono and 3D-EB hiPSC-CM differentiation, we applied a Wnt signaling-modulated protocol to hiPSCs maintained as a master cell bank (MCB),^18^ which retained high pluripotency as indicated by greater than 90% SSEA4 positivity [Fig. 1(a) and supplementary material Fig. 1(A)]. The protocol incorporated the identical small chemical cues for 15 days without additional growth factors, differing only in the initial culture configuration: 2D monolayer differentiation (2D-Mono) in Geltrex-coated 12-well plates vs 3D embryoid body differentiation (3D-EB) in a suspension bioreactor that provided dynamic adaptations to changes in oxygen, pH, and temperature, with real-time monitoring of key parameters [Figs. 1(a) and 1(b)].

The 3D-EB protocol has been previously validated across multiple independent hiPSC lines, demonstrating high cardiomyocyte purity and reduced batch-to-batch variability.^18^ To isolate differentiation format as the sole variable in the present benchmarking study, we used a single hiPSC line (WTC-Cas9) and performed at least 3 independent differentiation batches per condition. Structural and functional measurements were obtained from multiple microtissues across batches.

Bright-field imaging revealed distinct morphological phenotypes between the two hiPSC-CM differentiation formats [Fig. 1(b)]. In 2D-Mono, cell confluency serves as a key morphological indicator of differentiation readiness, whereas in 3D-EB, EB diameter is used to monitor aggregate growth and determine the timing of CHIR99021 administration.^18^ For our hiPSC line, differentiation was initiated at an empirically optimized confluency of 57.5% at day 0 in 2D-Mono, and when EB diameter exceeded 100 *μ*m in 3D-EB [Figs. 1(c) and 1(d)]. In 2D-Mono, cells reached 100% confluence by day 2, indicating rapid surface expansion and limited space for further proliferation thereafter [Fig. 1(c)]. In contrast, 3D-EB grew within the bioreactor, increasing diameter from 128.6 *μ*m at day 0 to 210.9 *μ*m at day 2, and 247.5 *μ*m at day 4, representing a ∼1.9-fold increase from the initial size [Fig. 1(d)].

This difference in culture environment translated into a marked difference in cell yield under equivalent media conditions. When normalized to 100 ml of culture medium, 2D-Mono produced 67.1 × 10^6^ cells, whereas 3D-EB yielded 181.8 × 10^6^ cells, approximately a ∼2.7-fold increase [Fig. 1(e)]. Despite these differences in scalability, both formats generated more than 80% cTnT^+^ cardiomyocytes, suggesting comparable cardiac differentiation efficiency and purity [Fig. 1(f) and supplementary material Fig. 1(B)].

Interestingly, spontaneous beating frequency differed between the two systems. 2D-Mono exhibited an average rate of 1.15 Hz, with substantial batch-to-batch and well-to-well variability, whereas 3D-EB displayed a slower but less variable beating frequency of 0.59 Hz [Fig. 1(g), supplementary material Fig. 1(C), and Movie 1]. The lower spontaneous automaticity observed in 3D-EB suggests reduced intrinsic excitability compared with 2D-Mono, a feature that is often associated with less arrhythmogenicity.^23,24^ This difference is also relevant for downstream applications, because high spontaneous activity in wild-type hiPSC-CMs can complicate disease modeling or pharmacological testing by mimicking proarrhythmic behavior.^25,26^

The scalability and reproducibility advantages of 3D-EB differentiation observed here are not limited to the specific stirred-tank bioreactor system used in this study. The same Wnt-modulated protocol has been successfully implemented in simpler suspension systems, including magnetically stirred spinner flasks, while maintaining high cardiomyocyte purity and reduced batch-to-batch variability.^18^ Similar trends have been reported across independent EB-based and suspension differentiation approaches, which consistently demonstrate improved yield and reproducibility compared to 2D monolayer methods.^19,27^

Collectively, these results demonstrate that although 2D-Mono and 3D-EB differentiation protocols generate cardiomyocytes of comparable purity, the 3D-EB supports greater yield and slower spontaneous beating with reduced variability, establishing a scalable foundation for large-scale tissue engineering and human-relevant disease modeling.

### Structural maturation of 2D-Mono- and 3D-EB-derived hiPSC-CMs compared to NRVMs

The structure and function of cardiac muscle depend critically on anisotropic tissue organization, in which cardiomyocytes align along a common axis and assemble laterally coupled parallel myofibrils to enable efficient force generation and coordinated contraction.^28–30^ To quantify these essential features of myocardial architecture in engineered tissues, we quantified established structural metrics, including sarcomere length and orientation order parameters (OOP), using F-actin OOP to measure cytoskeletal alignment and sarcomeric OOP to evaluate local Z-disk organization from α-actinin striation coherence.^2,31–33^ In addition, because the cytoskeleton mechanically couples the extracellular matrix to the nucleus, nuclear orientation and eccentricity provide a sensitive readout of intracellular tension and mechanotransduction.^32–34^

**FIG. 2.**
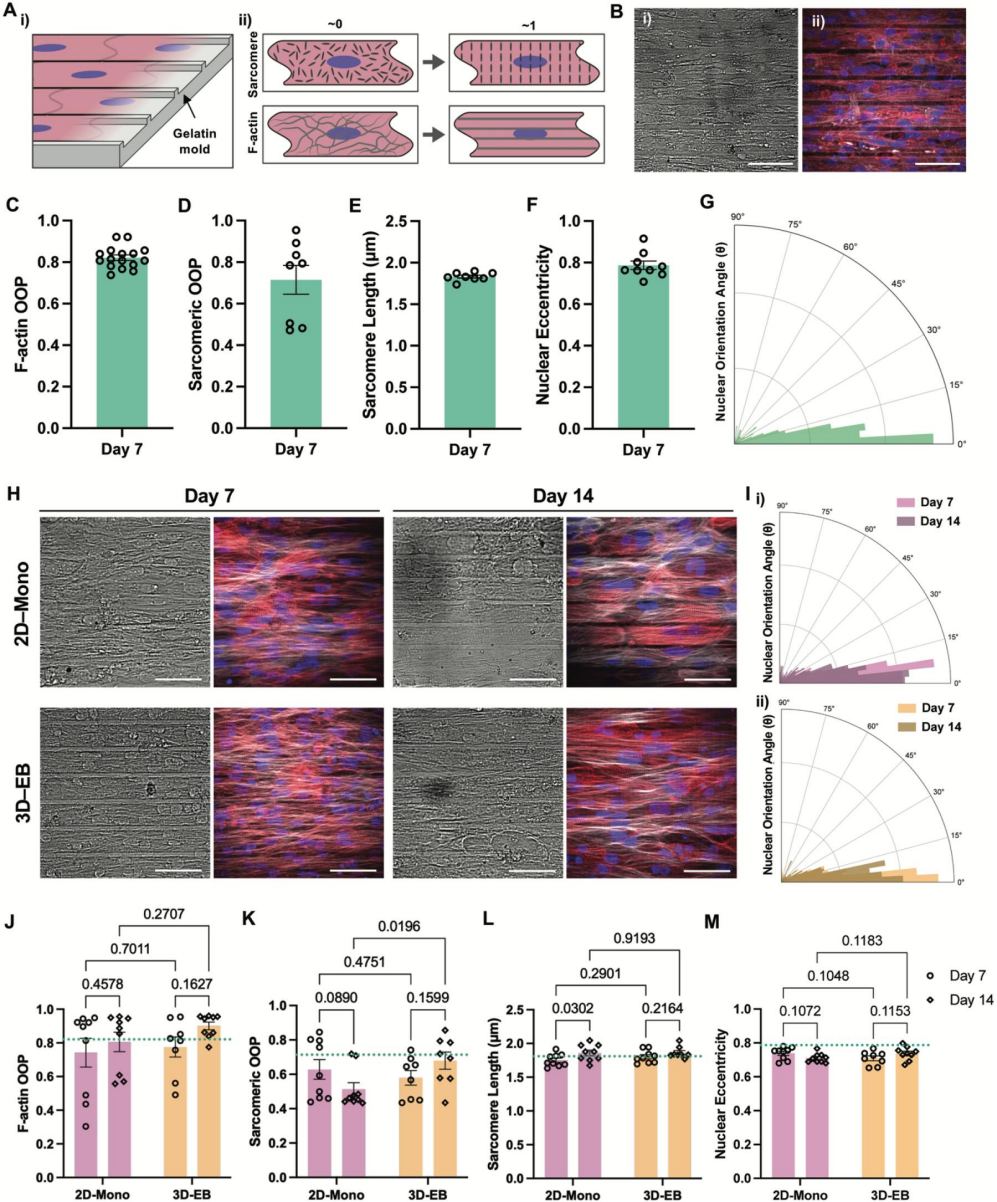
Structural phenotyping of engineered laminar cardiac tissues. (a) Schematic of anisotropic laminar tissue formation using gelatin molds (ridge width 25 *μ*m, groove width 4 *μ*m, and groove depth 5 *μ*m) (i) and quantification of alignment by OOP (ii). (b) Representative bright-field and immunofluorescence images of NRVM tissues at day 7 stained for α-actinin (red), F-actin (white), and nuclei (blue). (c)–(g) Quantification of F-actin OOP, sarcomere OOP, sarcomere length, nuclear eccentricity, and orientation. (h) Representative images of micromolded hiPSC-CM tissues from 2D-Mono and 3D-EB cultures at days 7 and 14. (i)–(m) Comparative analysis of myofibrillar and nuclear organization in 2D-Mono and 3D-EB tissues on days 7 and 14. Green dashed lines indicate the mean NRVM values: 0.821 (j), 0.714 (k), 1.81 (l), and 0.787 (m). Scale bar: 50 *μ*m in (b) and (h). Values are presented as mean ± SEM. Statistical significance was evaluated by two-way ANOVA; *P* values are indicated above each comparison. Each dot represents one microtissue for (c)–(e) [*n* = 16 for (c); *n* = 8 for (d) and (e); *n* = 9 for (f) from 4 independent harvests] and for (j)–(m) (2D-Mono D7: 9, D14: 9; 3D-EB D7: 8, D14: 9 from 3 different batches).

To this end, we examined whether 2D-Mono- and 3D-EB-derived hiPSC-CMs differed in their structural organization when assembled into anisotropic laminar tissues (Fig. 2). All cells were seeded onto micromolded gelatin substrates featuring 25 *μ*m ridges, 4 *μ*m grooves, and 5 *μ*m height, with a Young's Modulus of 55.6 kPa, which is designed to promote long-term culture and anisotropic alignment [Fig. 2(ai), Movie 2].^35,36^ This substrate is identical to that used in our G-node chip (for Ca^2+^V measurement) and MTF (for contractility measurement) platforms,^22,35,36^ enabling direct comparison of structural and functional properties under matched microenvironmental conditions.

Myofibrillar organization was evaluated by immunostaining for F-actin (white), α-actinin (red), and nuclei (blue) and quantified using the OOP method [Figs. 2(aii) and 2(b)]. NRVM tissues displayed highly ordered myofibrillar structures aligned along the microgroove direction, with F-actin OOP = 0.82, sarcomeric OOP = 0.72, and sarcomere length = 1.83 *μ*m [Figs. 2(c)–2(e)]. NRVM nuclei were elongated [eccentricity >0.787, Fig. 2(f)] and oriented parallel to the ridge axis [Fig. 2(g)], consistent with prior findings that anisotropic boundary conditions align cardiomyocyte nuclei along the maximal principal stress axis.^32,33,37^

Both 2D-Mono and 3D-EB tissues aligned with the micromolded substrate cue and formed discernible sarcomeric striations, with nuclei oriented parallel to the ridge axis at both days 7 and 14 [Figs. 2(h) and 2(i)]. F-actin OOP values were similar across all groups (2D-Mono: day 7, 0.74; day 14, 0.81; 3D-EB: day 7, 0.78; day 14, 0.9) and remained above 0.7 [Fig. 2(j) and supplementary material Fig. 2(A)], indicating global alignment guided by the substrate and confirming the formation of anisotropic tissues. Sarcomeric OOP values did not differ significantly across most conditions [Fig. 2(k) and supplementary material Fig. 2(B)], with a statistically significant difference observed only at day 14, where 2D-Mono exhibited lower values (0.51) compared with both 3D-EB (0.68) and NRVMs (0.71). Sarcomere length remained comparable across groups and time points at approximately 1.8 *μ*m [Fig. 2(l) and supplementary material Fig. 2(C)]. Nuclei eccentricity differed across groups, with statistically significant differences relative to NRVMs observed in 2D-Mono at day 14 and 3D-EB at day 7 [Fig. 2(m) and supplementary material Fig. 2(D)]. These differences indicate a relatively rounder morphology under these conditions, which has been associated with reduced cytoskeletal prestress and incomplete mechanical coupling.^33^ Overall, hiPSC-CMs and NRVMs exhibited comparable structural organization across most measured parameters, with differences limited to specific conditions.

Prior work suggests that such myofibrillar discontinuity is often accompanied by less organized costamere structure and reduced integrin-based force transmission at the membrane, providing a mechanistic basis for the lower cytoskeletal prestress observed in hiPSC-CM tissues.^38,39^ Consistent with this, the decreased tension transmitted through the actin–titin–LINC axis results in rounder, less elongated nuclei in hiPSC-CMs.^40,41^ Moreover, as the gelatin hydrogel substrate used here (ridges/grooves, 55 kPa modulus) promotes alignment but does not fully replicate adult myocardial stiffness or extracellular matrix (ECM) composition, hiPSC-CMs may maintain a more compliant nuclear envelope and less condensed chromatin than primary myocytes, preventing nuclear deformation in these engineered tissues.^33,42^

Together, these results indicate that 3D-EB tissues exhibit higher sarcomeric organization than 2D-Mono at day 14. At this time point, 3D-EB tissues showed no statistically significant differences from NRVMs in either sarcomeric organization or nuclear eccentricity, whereas 2D-Mono remained significantly different. These findings suggest that the structural gap relative to NRVMs is substantially reduced in 3D-EB tissues.

### Functional maturation assessed by calcium wave velocity

Coordinated calcium handling is essential for synchronized contraction in cardiac tissue and serves as a key indicator of functional maturation.^26^ Uniform and rapid calcium wave transmission reflects effective cell–cell coupling and integration, whereas a slower or heterogeneous wave indicates immature or poorly connected networks.^43^ Because Ca^2+^V integrates both cellular electrophysiology and multicellular connectivity, it provides an indicator of how differentiation strategy and tissue organization influence functional performance.

**FIG. 3.**
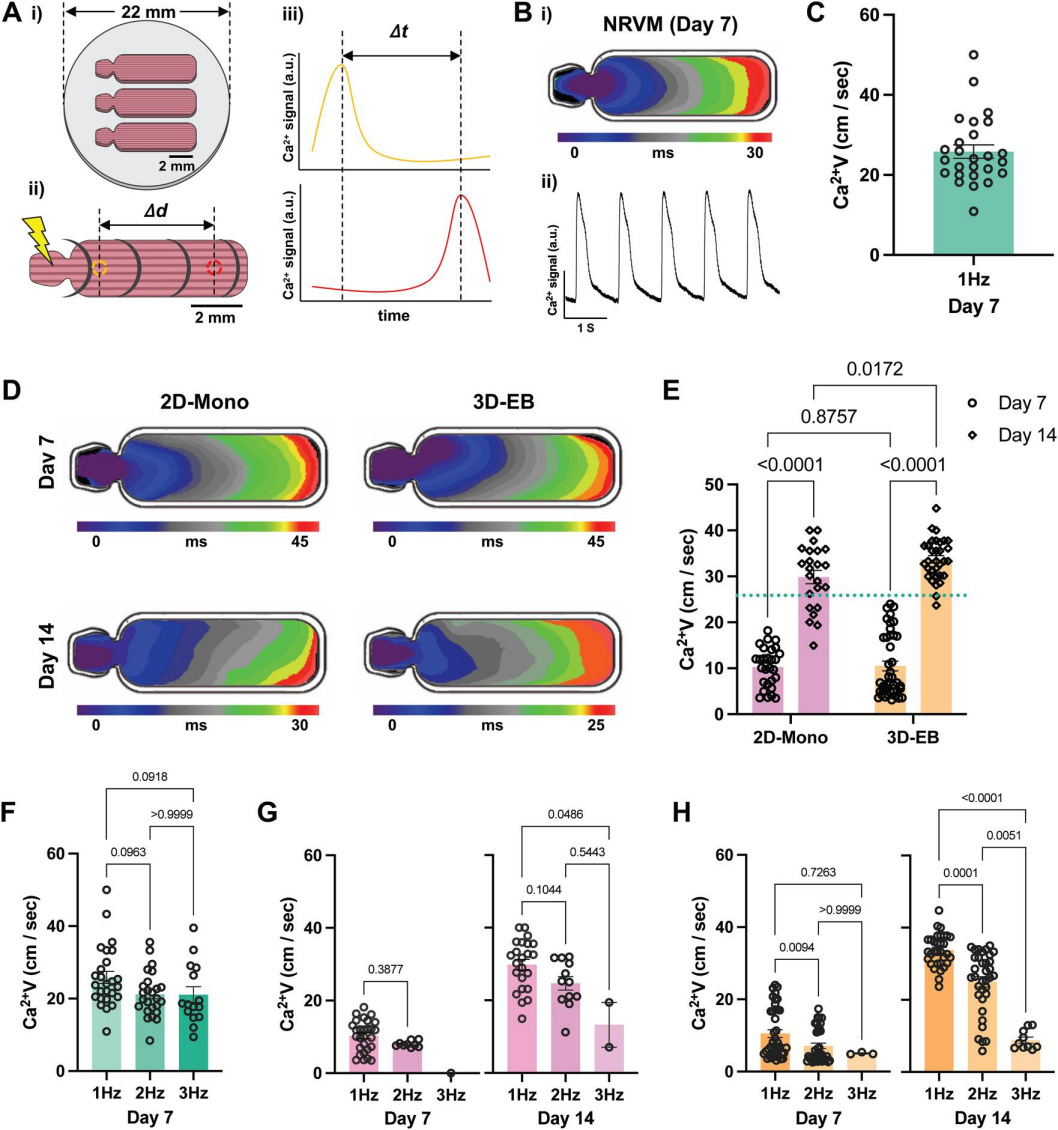
G-node chips for assessment of calcium wave velocity at 1 Hz. (a) Schematic of the G-node chip design showing microtissue dimensions (i) and the analysis of longitudinal Ca^2+^V of calcium waves (ii) and (iii). (b) Representative isochrone maps (i) and calcium traces (ii) from NRVM G-node chips at day 7, and (c) quantification of mean Ca^2+^V. (d) Representative isochrone maps of hiPSC-CM G-node chips at two different culture time points. (e) Comparative analysis of Ca^2+^V in hiPSC-CMs, with the green dashed line indicating the NRVM mean value (28.88 cm/s). (f) Ca^2+^V measurements at 1–3 Hz pacing on day 7 of NRVM. Ca^2+^V measurements at 1–3 Hz pacing on days 7 and 14 for 2D-Mono G-node tissues (g) and 3D-EB G-node tissues (h). Statistical significance was evaluated using two-way ANOVA for (e) and Kruskal–Wallis test for (f)–(h); *P* values are indicated above each comparison. Each dot represents one microtissue: *n* = 26 (c); 1 Hz: 26, 2 Hz: 25, 3 Hz: 15 (f) from 6 independent harvests; 2D-Mono day 7, 1 Hz: 28, 2 Hz: 9, 3 Hz: 0 from 5 different batches; 2D-Mono day 14, 1 Hz: 23, 2 Hz: 12, 3 Hz: 2 from 4 different batches (g); 3D-EB day 7, 1 Hz: 41, 2 Hz: 40, 3 Hz: 3 from 8 different batches; and 3D-EB day 14, 1 Hz: 32, 2 Hz: 35, 3 Hz: 10 from 6 different batches (h). Values are shown as mean ± SEM.

To quantify these dynamics, we adapted our previously described G-node (geometrically insulated node) in which a ∼1700-cell pacemaking region modeled the geometric constraint of the sino-atrial node that drives action potential wave propagation directly by an engineered source–sink balance.^36^ Using this principle, we designed a cardiac tissue chip containing three independent microtissues, each incorporating its own G-node region on a circular coverslip [Fig. 3(ai)]. A point stimulus was applied to each G-node to initiate a unidirectional signal wave toward the distal edge of the corresponding tissue [Fig. 3(aii)]. Optical mapping following calcium dye staining was used to quantify longitudinal Ca^2+^V and pacing capture, with Ca^2+^V calculated as the ratio of wave distance (*Δd*) to activation time difference (*Δt*) [Figs. 3(aii) and 3(aiii)].

In NRVM tissues, 1 Hz stimulation is shown as a representative condition, producing clear isochronal maps and corresponding calcium-transient traces [Figs. 3(bi) and 3(bii)]. The average Ca^2+^V at day 7 was 25.8 cm/s across 26 microtissues [Fig. 3(c), Movie 3], which aligns with previously reported Ca^2+^Vs for engineered NRVM monolayers and microtissues (20–35 cm/s), confirming that our platform can reproduce physiologically relevant calcium wave speeds.^44,45^

Both 2D-Mono and 3D-EB tissues exhibited substantial Ca^2+^V increases from days 7 to 14, demonstrating time-dependent functional maturation. Ca^2+^V rose from 10.3 cm/s to 29.9 cm/s in 2D-Mono and from 10.5 to 33.7 cm/s in 3D-EB tissues with representative isochronal maps illustrating calcium waves [Figs. 3(d) and 3(e), supplementary material Fig. 3(A), and Movie 3]. Unlike NRVMs, which maintained stable Ca^2+^V at increased pacing frequencies [Fig. 3(f)], both hiPSC-CM tissues showed reduced Ca^2+^V at higher pacing rates [Figs. 3(g) and 3(h)]. Pacing-capture analysis across 1, 2, and 3 Hz stimulation revealed improved capture performance at day 14 in both hiPSC-CMs, with 3D-EB tissues displaying slightly higher pacing fidelity than 2D-Mono [supplementary material Fig. 3(b)]. Together, these findings indicate that extended culture duration enhances calcium handling in hiPSC-CMs and that 3D differentiation provides an additional advantage in supporting more reliable high-frequency pacing.

We next examined spontaneous activation patterns by determining where calcium waves originated in the absence of stimulation. Each microtissue was divided into four regions (G-node, left corner, mid region, and right corner) to assign the initiation site [supplementary material Fig. 3(cii)]. NRVM tissues are predominantly activated from the G-node, whereas 2D-Mono and 3D-EB tissues frequently initiate activity from ectopic regions [supplementary material Fig. 3(ciii)]. This behavior likely reflects fundamental electrophysiological differences between cell types rather than a source–sink mismatch alone.^43^ hiPSC-CMs exhibit increased intrinsic automaticity due to elevated HCN4 expression and reduced IK1, resulting in a depolarized resting membrane potential and the capacity for spontaneous depolarization across the tissue.^46^ Under these conditions, regions with lower electrotonic load, such as distal free edges, can act as preferential sites of ectopic activation. In contrast, NRVMs maintain a more stable resting potential and stronger electrotonic sink, enabling consistent G-node-driven activation.^36^ Notably, the reproducible right-corner dominance observed at day 14 suggests additional spatial or geometric contributions that are not fully explained by source–sink considerations alone. While the present data clearly demonstrate this pattern, a definitive mechanistic explanation will require computational modeling that incorporates both hiPSC-CM electrophysiology and G-node geometry.^47^ Such approaches may help determine whether reduced electrotonic loading at tissue boundaries or heterogeneity in automaticity contributes to the observed spatial bias.

The reduced ability of hiPSC-CMs to maintain Ca^2+^V at higher pacing frequencies, in contrast to the stable Ca^2+^V observed in NRVMs, likely reflects their immature calcium-handling machinery. hiPSC-CMs exhibit slower SERCA-mediated calcium reuptake, reduced sarcoplasmic reticulum capacity, and immature T-tubule organization,^38,48,49^ all of which can lead to calcium-cycling fatigue during rapid stimulation. Stable high-frequency conduction requires coordinated regulation of calcium-handling proteins and ion-channel expression, which continue to lag behind NRVM levels. Prior studies have shown that metabolic and electrophysiological maturation, rather than structural alignment alone, drives suppression of spontaneous activity and supports reliable pacing capture, and that specialized maturation media can significantly enhance these properties.^50^ These considerations explain why hiPSC-CMs, even when structurally organized, remain prone to pacing-dependent CV reductions and ectopic activation.

Despite these limitations, the functional differences between the platforms are relevant for arrhythmia studies. Specifically, 2D-Mono tissues exhibited higher and more variable spontaneous beating rates [Fig. 1(g) and supplementary material Fig. 1(c)] and lower pacing-capture reliability at 2–3 Hz, whereas 3D-EB tissues maintained more stable 1:1 capture [Fig. 3(h) and supplementary material Fig. 3(B)]. These properties influence the ability to analyze conduction block, spiral wave initiation, and patient-specific arrhythmia mechanisms.^25,26,51^

### Functional maturation assessed by contractility

**FIG. 4.**
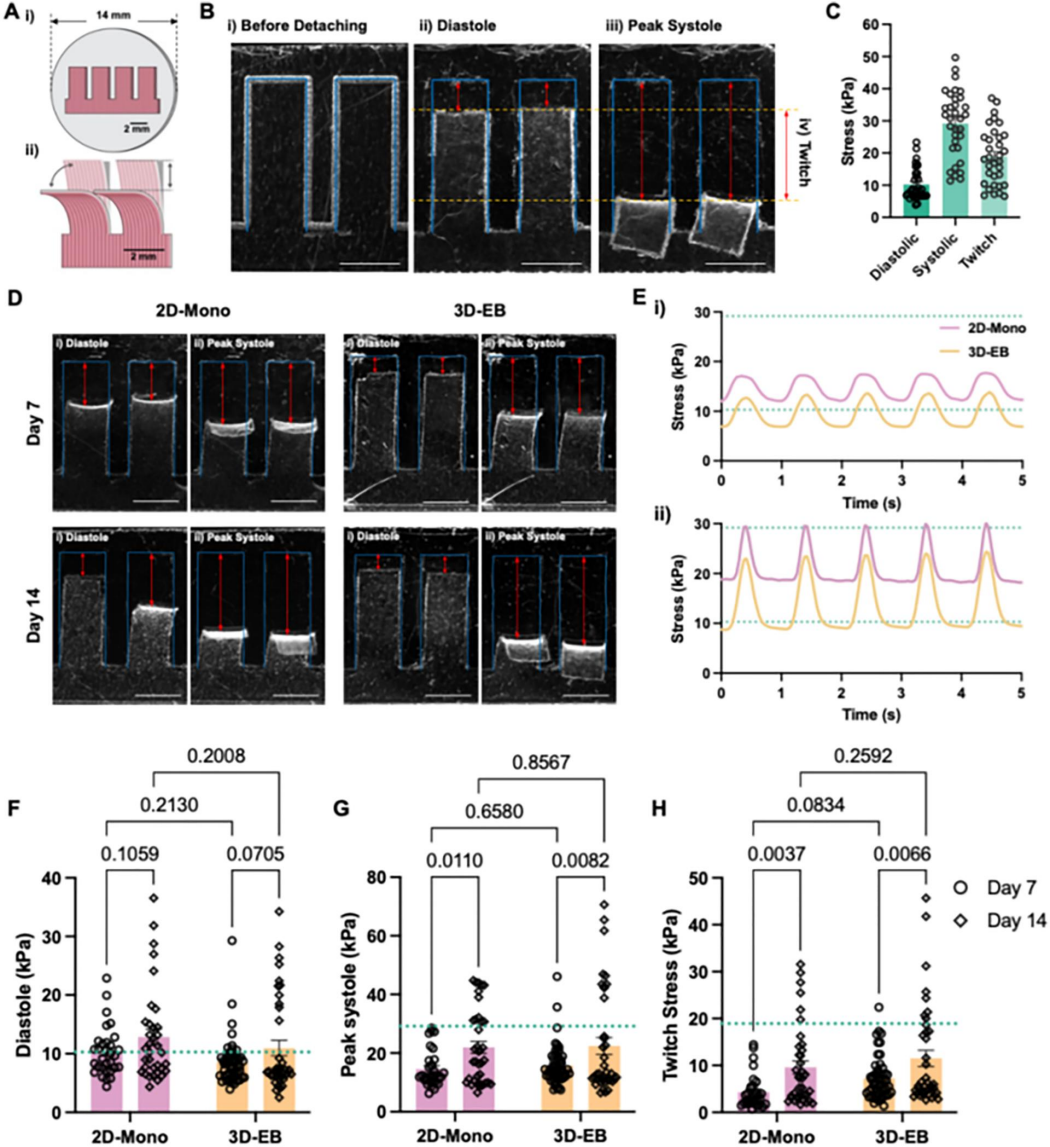
Muscular thin films (MTFs) for assessment of contractile stress at 1 Hz. (a) Schematic of the MTF platform showing cantilever dimensions (i) and the principle of stress quantification during contraction (ii). (b) Representative images of MTFs with NRVMs before detachment (i) and during diastole (ii) and peak systole (iii). (c) Quantification of contractile stress in NRVM tissues. (d) Representative images of MTFs with hiPSC-CMs at days 7 and 14 during diastole (i) and systole (ii). The blue line marks the original cantilever position prior to contraction, the red arrows indicate the deflection distance at diastole and systole, and the yellow dashed line denotes the net deflection between diastole and systole, which is used to compute twitch stress. (e) Representative stress traces for hiPSC-CMs at day 7 (i) and day 14 (ii), benchmarked against NRVM diastolic and systolic values (green dashed line). (f)–(h) Quantification of diastolic, systolic, and twitch stress in hiPSC-CMs at two time points, compared with NRVM reference values [green dashed line: 10.27 kPa for (f), 29.16 kPa for (g), and 18.92 kPa for (h)]. Scale bar: 2 mm in (b) and (d). Values are presented as mean ± SEM. Statistical significance was evaluated using two-way ANOVA; *P* values are indicated above each comparison. Each dot represents one cantilever: *n* = 33 from 4 independent harvests [NRVM (c)]; *n* = 31 from 5 different batches (2D-Mono D7), 37 from 4 different batches (2D-Mono D14), 52 from 6 different batches (3D-EB D7), and 39 from 4 different batches (3D-EB D14) for (f)–(h).

Beyond calcium handling, the ability of cardiac tissue to generate and transmit mechanical force represents an important integrative indicator of functional maturity. Contractile performance reflects not only excitation–contraction coupling but also the underlying structural organization, stiffness, and load-bearing capacity of the myocardium, key properties that determine the heart's pumping efficiency.^29,52,53^ To measure contractility, we employed our MTF platform based on micromolded gelatin hydrogels configured in a cantilever geometry [Fig. 4(a)]. This system mimics the anisotropic alignment and mechanical compliance of the ventricular myocardium, which is composed of aligned, elongated cardiomyocytes that coordinate contraction and transmit force efficiently.^22,35,54^ The MTF platform thus provides a physiologically relevant and scalable method for quantitatively assessing the contractile performance of engineered cardiac tissues.

Contractility in the MTF assay was quantified across three parameters: diastolic stress, representing the passive prestress or resting tension of the tissue; peak systolic stress, indicating the maximum active stress generated during contraction; and twitch stress, defined as the difference between systolic and diastolic stress, reflecting the tissue's net contractile force output [Fig. 4(b)]. At day 7, NRVMs generated a diastolic stress of 10.3 kPa, a peak systolic stress of 29.2 kPa, and a resulting twitch stress of 18.9 kPa [Fig. 4(c), Movie 4]. Representative experimental images of 2D-Mono and 3D-EB tissues at diastole, peak systole, and twitch [Fig. 4(d)] demonstrated that these contractile states were similarly capable of being resolved in the human tissues, with the corresponding stress-time traces further confirming robust contraction–relaxation dynamics [Fig. 4(e)]. In diastole, both 2D-Mono and 3D-EB tissues exhibited stress levels comparable to NRVMs, whereas in systole, both formats generated significantly lower stresses than NRVM tissues at both days 7 and 14 [Figs. 4(f) and 4(g) and supplementary material Fig. 4(A)]. In comparison, 2D-Mono tissues exhibited a progressive increase in twitch stress from 4.3 to 9.6 kPa between days 7 and 14, whereas 3-EB tissues showed a rise from 7.2 to 11.5 kPa over the same period [Fig. 4(h), supplementary material Fig. 4(A), and Movie 4]. These data indicate that both formats undergo functional maturation with extended culture, while 2D-Mono and 3D-EB tissues exhibit comparable overall contractile stress that remains lower than NRVMs.

Taken altogether, our structural, electrophysiological, and contractility measurements reveal a coherent explanation for the contractile deficit in hiPSC-CM tissues. Structurally, hiPSC-CMs exhibited lower sarcomeric OOP than NRVMs despite similar F-actin alignment and sarcomere length, indicating that human cells can align globally but lack the higher-order Z-disk registration, continuous myofibrillar bundles, and it may show lack of mature costamere anchoring required for efficient force transmission. Electrophysiologically, hiPSC-CMs achieved NRVM-like conduction velocity at baseline, reflecting early maturation of calcium handling and electrical coupling, but showed pacing-rate-dependent slowing of Ca^2+^V that NRVMs did not, consistent with limited calcium cycling reserve and slower channel recovery. These structural and rate-dependent functional limitations aligned with the significantly reduced systolic and twitch stresses observed in both 2D-Mono and 3D-EB tissues, even though diastolic stress was comparable. Thus, while anisotropic patterning supports near-native Ca^2+^V, incomplete myofibrillar maturation and limited calcium-handling robustness collectively constrain the ability of hiPSC-CMs to translate electrical activation into NRVM-like contractile force.

Although NRVMs serve as the traditional *in vitro* reference, the ultimate benchmark remains adult human ventricular myocardium. Compared with adult human cardiomyocytes, which display sarcomere lengths of ∼2.0–2.2 *μ*m,^55^ longitudinal conduction velocities of ∼60–70 cm/s,^56,57^ and peak twitch stresses of ∼40–80 kPa,^58,59^ the hiPSC-CMs generated by both 2D-Mono and 3D-EB formats, as well as NRVMs on our platform, remain functionally immature.

Nevertheless, the ability of hiPSC-CMs to achieve organized structure, stable Ca^2+^V, and measurable force generation without additional maturation cues highlights substantial intrinsic potential that can be further enhanced through advances in maturation technologies. Prior studies show that controlled electrical pacing, especially with gradually increasing intensity, drives adult-like ultrastructure, T-tubule formation, oxidative metabolism, and positive force–frequency behavior in engineered cardiac tissues.^60^ Metabolic interventions that shift cells from glycolysis toward fatty-acid oxidation, as well as supplementation with thyroid and glucocorticoid hormones, have been shown to enhance mitochondrial maturation, SERCA/RYR2 upregulation, and development of functional T-tubule networks.^61–63^ Together, these approaches are known to enhance calcium handling and contractile output, suggesting that the hiPSC-CM populations generated here could reach substantially higher physiological maturity when integrated with such cues.

## CONCLUSIONS

Shifting from animal-based to human-specific cell sources represents a critical advancement in cardiac tissue engineering and biomedical research. While NRVMs have traditionally functioned as the reference standard, their rodent origin imposes inherent limitations on human and clinical applicability. In this study, we provide an integrated analysis of hiPSC-CMs within laminar microphysiological systems, combining anisotropic tissues on G-node chips with MTFs to assess structural, electrophysiological, and contractile function in direct comparison to NRVMs. The findings define internal reference standards that support a laboratory-wide transition from animal- to human-derived cardiac models, advancing reproducibility, ethical compliance, and translational relevance.

3D bioreactor-derived hiPSC-CMs demonstrated scalable production, improved sarcomeric organization, and enhanced Ca^2+^V compared to 2D monolayer-derived hiPSC-CMs. Although their contractile performance has not yet matched that of NRVMs, 3D-EB hiPSC-CMs achieved key structural and functional benchmarks across G-node and MTF platforms, establishing a validated path toward replacing NRVMs with fully human *in vitro* cardiac systems.

Importantly, all differentiation protocols used in this study relied solely on small-molecule modulation without additional selection or maturation steps. When combined with established maturation strategies such as metabolic conditioning, hormone supplementation, cell-type selection, or electromechanical stimulation, 3D-EB hiPSC-CMs are expected to reach even higher levels of physiological maturity. Together, these findings highlight 3D bioreactor-derived hiPSC-CMs as a scalable, reproducible, and human-relevant cell source for disease modeling, although biochemical or electromechanical maturation strategies will still be required to fully match primary cardiomyocyte performance.

## METHODS

### Neonatal rat ventricular myocyte (NRVM) isolation and culture

All procedures involving neonatal rat harvest were conducted in accordance with protocols approved by the Institutional Animal Care and Use Committee (IACUC) at Harvard University. Neonatal rat ventricular myocytes (NRVMs) were isolated from 2-day-old Sprague–Dawley rat pups (Charles River Laboratories, Wilmington, MA) as previously described.^21,22^ Briefly, ventricles were dissected under sterile conditions, minced, and digested overnight in 1 mg/ml trypsin at 4 °C with gentle agitation, followed by 1 mg/ml of collagenase II (#LS004177, Worthington Biochemical Corp., Lakewood, NJ) dissociation at 37 °C with several rounds of trituration. The resulting cell suspension was filtered through a 40 *μ*m strainer, centrifuged, and pre-plated twice (45 min each) to reduce fibroblast contamination. Purified cardiomyocytes were cultured in Medium 199 (#1150-067, Thermo Fisher Scientific, Waltham, MA) supplemented with 10% fetal bovine serum (FBS), 3.5 g/l glucose, 10 mM HEPES (#15630-080, Thermo Fisher Scientific), 2 mM L-glutamine, and standard additives. After 48 h, the FBS concentration was reduced to 2%. Cultures were maintained at 37 °C in a humidified 5% CO_2_ incubator.

For each isolation, ventricles were pooled from a full litter of 10 neonates. All NRVM structural and functional data reported here were generated from 3 to 6 independent isolations, each from a separate litter, representing a total of approximately 30–60 neonatal animals.

### Human-induced pluripotent stem cell expansion

The hiPSC line WTC-Cas9 was derived from the WTC-11 parental line (#GM25256, Coriell Institute, Camden, NJ) by inserting a CAG-rtTA:TetO-Cas9 cassette into the AAVS1 locus, and a master cell bank (MCB) was established from this line as previously described.^18^ hiPSCs were cultured as 2D monolayers in Essential 8™ (E8; #A1517001, Thermo Fisher Scientific) medium with supplement on Geltrex™ (#A1413302, Thermo Fisher Scientific)-coated (1:100 in PBS, ≥2 h at 37 °C) 6-well plates. For passaging, cells were washed with PBS and dissociated with Versene (Thermo Fisher Scientific) for 5 min at 37 °C, then resuspended in E8 medium containing 10 *μ*M Y-27632 (#72304, STEMCELL Technologies, Vancouver, Canada) to enhance viability. The medium was replaced the following day with E8 without a ROCK inhibitor. Cultures were maintained at 37 °C, 5% CO_2_ with daily medium changes and passaged at 70%–80% confluency.

### 2D-Mono and 3D-EB hiPSC-CM differentiation

Human-induced pluripotent stem cells (hiPSCs) were differentiated into cardiomyocytes using a small-molecule Wnt modulation protocol adapted from previously described methods.^18,64^ For 2D-Mono differentiation, hiPSCs were cultured in 12-well plates in E8 medium and induced when confluency reached 50%–60%. For 3D-EB differentiation, hiPSCs were expanded in three T75 flasks, harvested, and 50 × 10^6^ hiPSCs were inoculated into DASGIP® stirred-tank bioreactors (Eppendorf) (100 ml E8 medium, 60 rpm, 100% dissolved oxygen, pH 7.0, 37 °C) to form embryoid bodies (EBs), and differentiation was initiated once EBs reached 100–300 *μ*m in diameter.

On differentiation day 0 (dd0), cultures were switched to RPMI 1640 (#61870036, Thermo Fisher Scientific) with B27 minus Insulin (#17504044, Thermo Fisher Scientific) and 7 *μ*M CHIR99021 (#72054, STEMCELL Technologies). Medium was replaced with RPMI/B27 minus Insulin on day 1 (dd1), followed by 5 *μ*M IWR-1-endo (#72564, STEMCELL Technologies) treatment on day 2 (dd2) for 48 h. On day 4 (dd4), cultures returned to RPMI/B27 minus Insulin. Spontaneous contractions typically appeared by day 6 (dd6). Beginning on day 8 (dd8), cells were maintained in RPMI/B27 with Insulin with media changed every 2 days until day 15.

On day 15, 2D-Mono was dissociated with 300 U/ml of collagenase (#LS004176, Worthington Biochemical Corp.) (30 min in incubator), filtered (70 *μ*m), and cryopreserved in STEMdiff™ Cardiomyocyte Freezing Medium (#05030, STEMCELL Technologies) (5–10 × 10^6^ cells/vial) using a Mr. Frosty™ container prior to liquid-nitrogen storage. On day 15, EBs were dissociated in collagenase (3–4 h, 37 °C), filtered (70 *μ*m), and cryopreserved identically to 2D-Mono.

### Thawing and seeding hiPSC-CMs

Neonatal rat ventricular myocytes (NRVMs) were seeded immediately after isolation without cryopreservation. Frozen hiPSC-CMs were thawed rapidly at 37 °C and diluted dropwise into RPMI 1640 medium without B27 supplement containing 10 *μ*M Y-27632 to a final volume of 10–15 ml. The suspension was centrifuged at 200 × g for 5 min, and the pellet was resuspended in RPMI/B27 medium supplemented with 10 *μ*M Y-27632.

Before seeding, G-node chips and muscular thin films (MTFs) were coated with an extracellular matrix mixture of fibronectin (20 *μ*g/ml, #356008, Corning, NY) and Geltrex (1:100 in PBS) for 2 h at 37 °C or overnight at 4 °C. Each G-node chip (three microtissues per chip, positioned in a 12-well plate) was seeded with 2–3 × 10^6^ cells/chip, whereas each MTF substrate (four cantilevers per chip, positioned in a 24-well plate) was seeded with 1 × 10^6^ cells/chip.

### Flow cytometry for pluripotency and cardiomyocyte purity

Flow cytometry was used to assess pluripotency in undifferentiated hiPSCs (SSEA4, stage-specific embryonic antigen-4; #130-123-815, Miltenyi Biotec, Charlestown, MA) and cardiomyocyte purity after differentiation (cTnT, cardiac troponin T; #130-119-575, Miltenyi Biotec) using an identical sample-preparation workflow. Approximately 1 × 10^6^ cells were collected for each assay—hiPSCs were sampled immediately before 2D-Mono seeding or 3D bioreactor inoculation for SSEA4, and hiPSC-CMs were sampled on day 15 of differentiation for cTnT analysis, respectively. Cells were washed with 1:10 diluted BD Perm/Wash™ buffer (BD Biosciences, San Jose, CA), centrifuged at 200 × g for 5 min, fixed in 500 *μ*l BD Cytofix™ for 10 min, washed, and resuspended in 196 *μ*l Perm/Wash™ buffer. Two 98 *μ*l aliquots were prepared per sample and stained overnight at 4 °C with either anti-cardiac troponin T (cTnT) or anti-SSEA4 antibodies (1:50), alongside corresponding isotype controls (#130-113-446, Miltenyi Biotec). The following day, samples were washed, resuspended in PBS, and analyzed on a Cytek Aurora spectral cytometer with daily QC using SpectroFlo® QC beads (Cytek Biosciences, Fremont, CA). Data integrity and gating were verified immediately after acquisition, and final analysis was performed using SpectroFlo™ or FlowJo. SSEA4 positivity (>90%) was required to confirm pluripotency before initiating differentiation, and only batches with >80% cTnT^+^ hiPSC-CMs were used for downstream experiments.

### Bright-field imaging and spontaneous beating analysis

Bright-field images of 2D-Mono and 3D-EB cultures during differentiation were acquired using an EVOS microscope (Thermo Fisher Scientific). Confluency in 2D-Mono cultures and EB diameter in 3D-EB cultures were quantified in ImageJ using threshold-based segmentation with manual verification. Spontaneous beating activity was quantified from bright-field videos recorded at 24 fps. A region of interest (ROI) was manually selected in each video to maximize detectable pixel-intensity changes, typically along the EB boundary in 3D-EBs or within a representative contracting region in 2D-Mono cultures. Mean pixel intensity was computed for each frame in MATLAB (MathWorks), and the resulting traces were smoothed to reduce noise. Negative peaks corresponding to contraction events were identified, and beating frequency (beats per minute, BPM) was calculated from the intervals between successive peaks.

### G-node chip and muscular thin film (MTF) substrate fabrication

Fabrication followed a previously published protocol.^36,65^ Acrylic substrates for both G-node chips and muscular thin films (MTFs) were produced from 1-mm-thick acrylic sheets (McMaster-Carr) covered with two layers of laboratory labeling tape (VWR, Radnor, PA). G-node substrates were laser-patterned into 20-mm-diameter circles containing three rectangular wells (2.2 × 7 mm) connected to a 1-mm pacing node, while MTF substrates were cut into 15.6-mm-diameter circles by removing the inner tape region to expose the cantilever area. For initial cleaning, all substrates were immersed in 10% bleach for 30 min, rinsed overnight in running de-ionized water, sonicated in 70% ethanol for 10 min, and dried under a biosafety hood for 1 h.

Micromolding was achieved using a gelatin–microbial transglutaminase (MTG) molding process. Gelatin (20% w/v; Type A, 175 Bloom, #G2625, Sigma-Aldrich) was dissolved in PBS at 65 °C until fully melted, then sonicated for 10 min to remove bubbles. MTG (8% w/v in PBS; #SKU:1002, Activa TI) was warmed to 37 °C and degassed in a desiccator. Equal parts of gelatin and MTG stock solutions were mixed immediately before use to generate a final solution of 10% w/v gelatin and 4% w/v MTG, which forms a cured gel with a stiffness of approximately 55.6 kPa.^35^ The solution (≈0.25 ml) was rapidly pipetted onto the exposed rectangular regions of each acrylic substrate. Line-patterned PDMS stamps (ridge width 25 *μ*m, groove width 4 *μ*m, and groove depth 5 *μ*m) were inverted onto the gelatin layer, a 200 g weight was applied to ensure uniform molding, and the samples were covered in a glass jar and allowed to cross-link overnight at room temperature. After curing, substrates were immersed in PBS for 1 h to rehydrate the gelatin, and PDMS stamps were carefully removed.

For MTF fabrication, the micromolded gelatin layer was then laser-engraved to create four cantilevers (2 × 5 mm^2^) using an Epilog CO_2_ laser system (power 5%, speed 9, and frequency 2500 Hz).

All patterned substrates were rinsed, stored in PBS, sterilized in 70% ethanol for 10 min, rinsed three times with PBS, and coated with fibronectin (20 *μ*g/ml) and Geltrex (1:100 in PBS) for 2 h at 37 °C or overnight at 4 °C before cell seeding.

### Immunostaining

Cells were fixed in 4% paraformaldehyde for 10 min at room temperature, washed with PBS, and permeabilized with 0.1% Triton X-100 for 10 min at room temperature. Samples were then blocked with 3% bovine serum albumin (BSA) for 1 h to minimize nonspecific binding. After three PBS washes, cells were incubated overnight at 4 °C with the primary antibody anti-sarcomeric α-actinin (1:400 dilution, ab68167, Abcam) diluted in 1% BSA. The following day, samples were washed thoroughly with PBS and incubated with Alexa Fluor™ 488-conjugated secondary antibody for sarcomeric α-actinin (1:400, #A11008, Thermo Fisher Scientific), Alexa Fluor™ 647-phalloidin (1:400, Thermo Fisher Scientific) to label F-actin, and Hoechst 33342 (1:500, Life Technologies) for nuclear staining. Samples were mounted with ProLong™ Diamond Antifade Mountant (Invitrogen), allowed to cure, and stored at 4 °C prior to imaging. Confocal images were acquired on an Andor spinning-disk system mounted on an Olympus IX83 microscope (Tokyo, Japan) equipped with 20× to 40× objectives.

### Orientation order parameter analysis for structural phenotyping

Myofibrillar organization was quantified using previously described methods with minor modifications.^31^ Immunostained images were preprocessed in ImageJ/Fiji using the OrientationJ plugin, and structural parameters were computed using a custom MATLAB script. The orientational order parameter (OOP) was used to assess global alignment across entire images rather than within user-defined regions of interest. F-actin OOP was calculated from images acquired at 20× magnification, whereas sarcomere OOP and sarcomere length were measured from α-actinin-stained images acquired at 40× magnification.

### Nucleus orientation and eccentricity analysis

Nuclear alignment and eccentricity were quantified from Hoechst-stained images in ImageJ/Fiji. Images were converted to 8-bit, thresholded, and processed with hole filling, despeckling, and watershed segmentation to isolate individual nuclei. Orientation and eccentricity values were obtained from ellipse fits using the “Analyze Particles” function, and incorrectly segmented or partial nuclei were excluded. Orientation distributions were subsequently plotted and analyzed in Python using custom scripts, and all measurements were aggregated across microtissues for each condition.

### Optical mapping in G-node chips

On days 7 and 14, G-node chips were incubated with 2 *μ*M X-Rhod-1 (#X14210, Thermo Fisher Scientific) dissolved in Pluronic™ F-127 (#P3000MP, Thermo Scientific) for 30 min, washed with culture medium, and transferred to the appropriate imaging solution: phenol red-free RPMI (#11835030, Thermo Fisher Scientific) B27 supplemented with 10 mM HEPES for hiPSC-CMs or Tyrode's solution (1.8 mM CaCl_2_, 5 mM glucose, 5 mM HEPES, 1 mM MgCl, 5.4 mM KCl, 135 mM NaCl, 0.33 mM of NaH_2_PO_4_, pH 7.4) for NRVMs. Cytoplasmic Ca^2+^ transients were recorded using a modified tandem-lens macroscope (Scimedia) equipped with a MiCAM Ultima high-speed camera, a plan APO 0.63× objective, a Lumencor collimator, and a 200 mW mercury lamp (X-Cite exacte). A Semrock filter set (580/14 nm excitation, 593 nm dichroic, and 641/75 nm emission) was used for X-Rhod-1 imaging. Videos were acquired at 400 fps while maintaining samples at 37 °C in a temperature-controlled dish. Electrical pacing was applied using a bipolar platinum electrode positioned at the distal end of the G-node, delivering 10 V, 20 ms pulses across the tested frequency range.

### Calcium wave velocity measurement

Only tissues that maintained 1:1 capture during pacing were included in the analysis, because loss-of-capture recordings do not allow reliable evaluation of velocity. Raw calcium imaging data were extracted from optical mapping videos using BrainVision software, restricted to a region of interest defined by a 7-pixel transverse window (1.17 mm) and a 25-pixel longitudinal window (4.12 mm). A 3 × 3 pixel spatial filter was applied to reduce noise. Calcium wavefronts were quantified using a custom MATLAB script. Ca^2+^V was calculated along the longitudinal axis based on the activation delay between the left and right edges of the analysis window [supplementary material Fig. 3(ci)]. Ca^2+^V values were further validated by manual measurement using a 5-mm distance (30 pixels) and the time difference between at least three distinct calcium upstroke peaks.

### Muscular thin film force measurements and quantifications

The gelatin MTFs were transferred to a 35 mm Petri dish and soaked in Tyrode's solution for NRVMs and RPMI 1640 medium with no phenol red with 10 mM HEPES for hiPSC-CMs. The dish was placed on the stage of a Zeiss Lumar.V12 stereomicroscope. Using fine tweezers, the excess gelatin and cell tissue surrounding the etched cantilevers were removed, and each cantilever gently peeled from the acrylic surface. The Petri dish was then transferred to a heating block to maintain a 37 °C temperature within the solution during imaging, and platinum field electrodes were introduced into the top of the dish such that they were immersed within the solution. The contracting MTF cantilevers were recorded at 30 fps using a Basler A601f-2 camera (Exton, PA) while pacing from 1 to 3 Hz at 20 V with a pulse waveform of 20 ms using a MyoPacer Cell Stimulator (IonOptix, Milton, MA).

To convert the gelatin cantilever deformation to stress, the *x*-projection of each cantilever was obtained by thresholding and binarizing the videos using image processing software (ImageJ, NIH). We assume that the deformed cantilever's shape corresponds to the arc of a circle, enabling the calculation of the curvature of the MTF from the *x*-projection, using the following algorithm, where *L*0 is the maximum length of the MTF, 5 mm, *r* is the radius of the fit circle, and *x* is the projection of the curled cantilever

$$\begin{array}{c} x=R\text{ sin}\left(\frac{L0}{R}\right),\;\text{if }x>\frac{L0}{\pi },\\ x=R,\;\text{if }x\leq \frac{L0}{\pi }. \end{array}$$

This accounts for both small and large deformations, with large cantilever deformation's fit circle radius corresponding to the *x*-projection. The curvature *K* is the inverse of the radius, *R* = 1/*K*. This is then converted into stress using a modified Stoney's equation, with *σ_c_* the stress, *t_b_*^2^ the tissue thickness, *t_f_* the gelatin film thickness, the gelatin Young's Modulus *Ē*, and *K* the curvature. We are then able to obtain diastolic and systolic stresses by averaging individual minima and maxima, respectively. Subtracting diastolic from systolic stress yields twitch stress, used to quantify MTF contractility,

$$\sigma_{K}=\bar{E}\frac{t_{b}^{2}}{6t_{f}}\frac{1}{\left(1+t_{f}/t_{b}\right)}K.$$

Gelatin thickness (185 *μ*m) was measured by embedding 200-nm Alexa Fluor 488 fluorescent beads (Invitrogen; stock 32.5 mg/ml) at a 1:4000 dilution into the gelatin before molding. Z-stacks were acquired on a Zeiss LSM 5 LIVE confocal microscope (20× objective) at two locations per chip across three chips. Stacks were resliced in ImageJ to obtain side-view profiles, and heights were measured manually. Tissue thickness (∼8 *μ*m) for NRVMs and hiPSC-CMs was measured using the same approach (supplementary material Fig. 4).

### Statistical analysis

Statistical analyses were performed using GraphPad Prism 10.0.2 (GraphPad Software). When comparing one variable, data were analyzed using the Mann–Whitney test or the Kruskal–Wallis test, followed by Dunn's *post hoc* test. Two-way ANOVA followed by Tukey's multiple comparisons test was applied when comparing two or more variables. Statistical tests used were denoted in the figure legends. The mean and standard error of the mean were plotted. *P* < 0.05 was considered statistically significant.

## SUPPLEMENTARY MATERIAL

See the supplementary material for additional figures and information concerning cell quality control and additional structural–functional readouts.

## Data Availability

The data that support the findings of this study are available from the corresponding author upon reasonable request.
